# A human MMTV-like betaretrovirus linked to breast cancer has been present in humans at least since the copper age

**DOI:** 10.18632/aging.103780

**Published:** 2020-07-31

**Authors:** Francesca Lessi, Nicole Grandi, Chiara Maria Mazzanti, Prospero Civita, Cristian Scatena, Paolo Aretini, Pasquale Bandiera, Antonio Fornaciari, Valentina Giuffra, Gino Fornaciari, Antonio Giuseppe Naccarato, Enzo Tramontano, Generoso Bevilacqua

**Affiliations:** 1 Division of Pathology, Department of Translational Research and New Technologies in Medicine, University of Pisa, Pisa, Italy; 2 Division of Paleopathology, Department of Translational Research and New Technologies in Medicine, University of Pisa, Pisa, Italy; 3 Division of Molecular Virology, Department of Life and Environmental Sciences, University of Cagliari, Cagliari, Italy; 4 Center for Anthropological, Paleopathological and Historical Studies of The Sardinian and Mediterranean Populations, Department of Biomedical Sciences, University of Sassari, Sassari, Italy; 5 Fondazione Pisana per la Scienza, Pisa, Italy; 6 Department of Laboratory Medicine, “San Rossore” Hospital, Pisa, Italy

**Keywords:** MMTV, HMTV, human betaretrovirus, breast cancer, cross-species transmission

## Abstract

The betaretrovirus Mouse Mammary Tumor Virus (MMTV) is the well characterized etiological agent of mammary tumors in mice. In contrast, the etiology of sporadic human breast cancer (BC) is unknown, but accumulating data indicate a possible viral origin also for these malignancies. The presence of MMTV*env*-like sequences (MMTVels) in the human salivary glands and saliva supports the latter as possible route of inter-human dissemination. In the absence of the demonstration of a mouse-man transmission of MMTV, we considered the possibility that a cross-species transmission could have occurred in ancient times. Therefore, we investigated MMTVels in the ancient dental calculus, which originates from saliva and is an excellent material for paleovirology. The calculus was collected from 36 ancient human skulls, excluding any possible mouse contamination. MMTV-like sequences were identified in the calculus of 6 individuals dated from the Copper Age to the 17^th^ century. The MMTV-like sequences were compared with known human endogenous betaretroviruses and with animal exogenous betaretroviruses, confirming their exogenous origin and relation to MMTV. These data reveal that a human exogenous betaretrovirus similar to MMTV has existed at least since 4,500 years ago and indirectly support the hypothesis that it could play a role in human breast cancer.

## INTRODUCTION

The etiology of the human breast carcinoma (BC) is unknown, with the exception of hereditary tumors (HBC) caused by the hereditary transmission of mutated tumor suppressor genes, such as BRCA1 and BRCA2 [1], and of the rare radiation-induced tumors. Estrogens are well known to be relevant promotional factors for breast carcinoma, but their transforming role has never been definitively demonstrated [2]. Non-hereditary BCs, which represent approximately 90% of BC, are called sporadic (SBC), which means occasional, to indicate the current lack of specific causes.

Over the last almost 50 years the hypothesis of a viral origin of sporadic BC has received increasing attention, being defined as “highly likely” in a very recent review by Lawson and Glenn [3]. Such hypothesis is based on the high similarity of BC to the experimental model of mammary tumors induced in mice by the betaretrovirus Mouse Mammary Tumor Virus (MMTV) [4] transmitted from mother to newborn by lactation [5]. In fact, this model has been very useful to clarify several aspects of BC biology, such as the concept of cancer progression, the recognition of the so-called preinvasive lesions, and the promotional role of estrogens [6].

While the first investigations suggesting the existence of a human mammary tumor virus date back to the seventies [7], only in 1995 the advent of molecular biology allowed to detect MMTV*env*-like sequences (MMTVels) in approximately 40% of infiltrating human breast carcinoma by Pogo and colleagues [8], who consequently introduced the term HMTV – human mammary tumor virus [9]. These data were later confirmed using laser capture microdissection and highly sensitive fluorescent PCR [10]. In contrast, it has been recently demonstrated that hereditary BC lacks MMTV sequences [1], as expected for the fact that HBCs, having a specific genetic etiology, do not need the action of a carcinogenetic viral agent. This result indirectly supports the reliability of the data in favor of the association between MMTV and sporadic breast carcinoma.

The hypothesis of a relationship between a human MMTV-like agent and BC raised the question of a potential zoonotic mouse-man transmission of MMTV. However, such a theory remained inconsistent with unconvincing sporadic reports of the possible transmission of MMTV from the mouse to humans directly or through animal vectors such as cat or dog [11–13]. Likewise, the possibility that human milk could be a mother-baby route of infection also appeared insufficiently supported [14, 15], as discussed later.

Subsequently, attention was focused on saliva – a common route of spreading of infectious diseases – and MMTVels were identified in human normal salivary glands and in saliva [16]. This finding, in the absence of sound data supporting mouse to man transmission of MMTV, suggested the existence of a human homologue of MMTV, possibly due to a MMTV mouse to man cross-species transmission that occurred in ancient times [17]. The demonstration of MMTVels in ancient individuals would strongly corroborate the hypothesis of a human MMTV-like betaretrovirus. To assess this possibility, we investigated the presence of MMTVels in the ancient dental calculus. The calculus, in fact, originates from saliva and is an excellent material for paleovirological studies.

Importantly, while no known human betaretroviruses are circulating in modern humans, the search for a human MMTV in human DNA can potentially be complicated by the presence of endogenous retroviruses (ERVs). ERVs are integrated sequences with retroviral origin that are present in our genome as well as in the genome of all vertebrates [18]. ERVs were acquired by a classical retroviral integration that occurred over millions of years ago within the genome of the host germ line cells, allowing their Mendelian inheritance through offspring. Human ERVs (HERVs), accounting for the 8% of the human genome, have accumulated mutations that generally compromised their coding potential, but several HERV groups have retained the capacity to be expressed and produce proteins. Some of the latter have been coopted during evolution for physiological functions, such as placenta development as well as the shaping of innate immunity pathways [19–21]. In addition, HERV expression is highly investigated for its possible contribution to human diseases, including autoimmunity and cancer [20, 22]. An updated general classification and characterization of approximately 3,200 HERV integrations in human genome reports the presence of 39 “canonical” HERV groups and 31 additional “non-canonical” groups of mosaic forms that arose from secondary integrations or recombination events [18]. Due to their similarity to exogenous retroviruses, HERV have also been broadly divided into three classes: Class I consists of gammaretrovirus-like and epsilon-like retroviruses, Class II of betaretrovirus-like retroviruses, and Class III of vaguely spumaretrovirus-like elements [18]. While no exogenous betaretroviruses are known to threaten humans nowadays, our genome harbors a betaretrovirus-like supergroup, namely HERV-K, as described in the early 80s when human sequences similar to the exogenous MMTV were found [23]. The HERV-K supergroup currently encompasses 10 distinct HERV groups, all sharing sequences similarities with MMTV. Consequently they were named HML (human MMTV-like) followed by a number from 1 to 10 [24].

The human genome harbors approximately 600 copies of HML sequences that share a significant level of intragroup identity [25]. Due to their abundant presence and to their remarkable similarity with MMTV, HML sequences can lead to misinterpretation when investigating the occurrence of exogenous betaretroviruses in humans, possibly leading to false positives.

This paper provides the first evidence that a MMTV-like betaretrovirus has been present in humans for thousands of years. The possibility of a contamination with murine material and endogenous betaretroviral sequences was carefully excluded.

## RESULTS

### MMTV*env*-like sequences are present in human remnants dated from the Copper Age to the 17^th^ century

**Figure 1:**
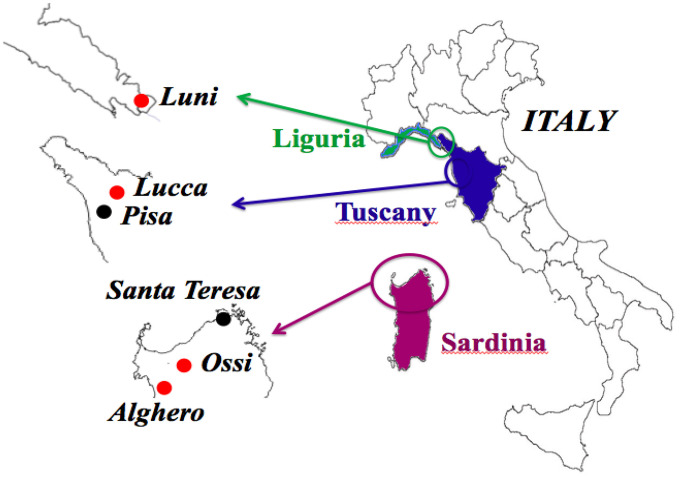
**Geographic areas of origin of the 36 skulls objects of the study.** Red dots indicate the four sites where the six cases positive for MMTV*env*-like sequences were identified: two in Ossi, Sardinia; two in Alghero, Sardinia; one in Luni, Liguria; one in Lucca, Tuscany. In four cases, the sequencing of both ENV1 and ENV 2 amplicons was accomplished; one of the ENV1 amplicons showed a C > T polymorphism, that did not cause an amino acid substitution: Ossi (ENV2), Luni (ENV1-C), Alghero (ENV1-C, ENV1-T). The two cases that dated back to the Copper Age came from Ossi, Sardinia.

**Figure 2:**
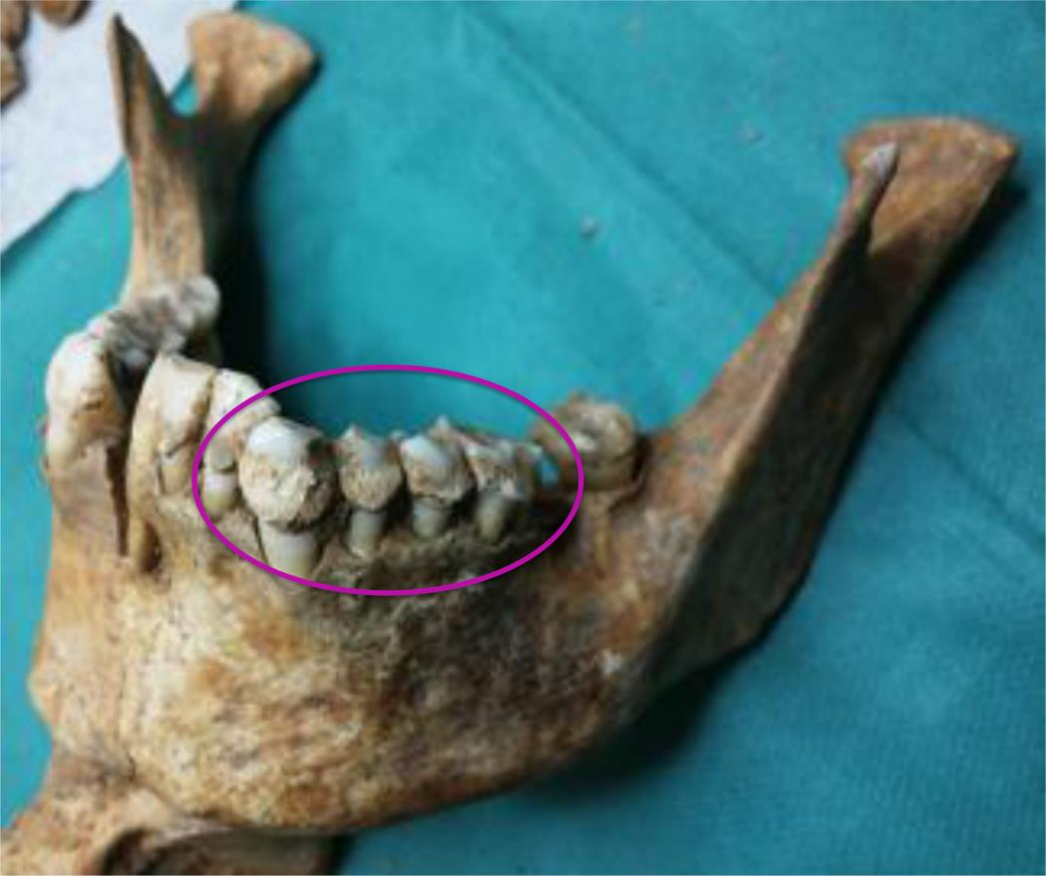
**A mandible of one of the skulls examined.** The circle indicates the abundance of calculus.

We had access to the skulls of 36 individuals dated from the Copper Age (details in Methods) to the 17^th^ century, all from Italian regions, namely, Sardinia, Tuscany and Liguria (Figure 1 and Table 1). The ante Christum (AC) cases, all from Sardinia, were buried in typical collective tombs from the Neolithic to the Bronze Age, named Domus de Janas (house of fairies) and Tomb of Giants. From Sardinia, additional samples were collected from a Roman necropolis of the Imperial Roman Age (1^st^-3^rd^ century), from a Medieval cemetery of the 14^th^-15^th^ century, and from a late Renaissance plague mass burial (1582-1583). Other skulls were from the Italian peninsula: from an early Medieval cemetery (6^th^-8^th^ century) in Liguria, a Medieval cemetery (12^th^-13^th^ century) in Tuscany (Pisa), and from the Guinigi’s family tomb of the Modern Age (15^th^-17^th^ century) again in Tuscany (Lucca). Dental calculus abundance (Figure 2) allowed the collection of specimens from all 36 cases and DNA extraction was successful in all cases. MMTVels were detected in six cases. Interestingly, the percentage of positivity (17%) was similar to that found in the saliva of healthy contemporary individuals [16].

**Table 1:** Characteristics of the individuals and molecular results.

**Site**	**Type of burial**	**Date**	**Cases and sex**	**Age**	**MMTVels positive**	**ENV 1**	**ENV 2**	**Sequencing**
S’Adde’e Asile, Ossi, Sassari, Sardinia	Domus de Janas	2712 ± 59 a.C.	3 2♂, 1♀	16 - 22	0			
Noeddale, Ossi, Sassari, Sardinia	Domus de Janas	2560 ± 51 a.C.	5 4♂, 1♀	20 - 29	2 ♂	2 ♂	1 ♂	ENV2 ♂
S’Isterridolzu, Ossi, Sassari, Sardinia	Domus de Janas	2070 ± 35 a.C.	4 1♂, 1♀, 2?	20 - 28	0			
La Testa, Santa Teresa, Sassari, Sardinia	Tomb of Giants	1200 ± 132 a.C.	3 3?	25 - 35	0			
Monte Carru, Alghero, Sardinia	Roman necropolis	1^st^-3^rd^ century	5 3♂, 2♀	24 - 34	0			
Amphitheatre, Luni, La Spezia, Liguria	early medieval cemetery	6^th^-8^th^ century	1 1♂	20 - 30	1 ♂	1 ♂	1 ♂	ENV1-C (♂)
Sant’Alessandro, Vecchiano, Pisa, Tuscany	medieval cemetery	12^th^-13^th^ century	5 1♂, 4♀	32 - 41	0			
San Michele, Alghero, Sardinia	late medieval cemetery	14^th^-15^th^ century	2 1♂, 1♀	32 - 43				
San Michele, Alghero, Sardinia	plague mass burial	1582-1583	6 3♂, 2♀, 1?	35 - 44	2 ♂♀	2 ♂♀		ENV1-C ♂ ENV1-T ♀
Guinigi Chapel, Lucca, Tuscany	family tomb	15^th^-17^th^ century	2 1♂, 1♀?	20 - >50	1 ♀?	1 ♀?		
total			36 17♂, 12♀, 7?		6 4♂/1♀/1♀?	6 4♂/1♀/1♀?	2 ♂	4 3♂/1♀

Dental calculus was obtained from the skulls of 36 individuals dated from the Copper Age to the 17^th^ century, all from Italian regions, namely Sardinia, Tuscany and Liguria. The a.C. cases were found in Domus de Janas and the Tomb of Giants, typical collective tombs from the Neolithic to the Bronze Age. Other Sardinian samples were from a Roman necropolis of the Imperial Roman Age, from a Medieval cemetery, and from a late Renaissance plague mass burial. Other skulls were from an early Medieval cemetery in Liguria, a Medieval cemetery in Tuscany and from the Guinigi’s family tomb of the Modern Age (15^th^-17^th^ century) in Tuscany. Six cases were positive for MMTVels. ENV1 was detected in all of them, two of which were also positive for ENV2. In particular, two cases were from Ossi, Sardinia, two from Alghero, Sardinia, one from Luni, Liguria, and one from Lucca, Tuscany. Four of the six positive cases were sequenced, three for ENV1 and one for ENV2; one of the ENV1 cases showed a C > T polymorphism, that did not cause an amino acid substitution. One of these six cases was found in Ossi (ENV2), one in Luni (ENV1-C), and two in Alghero (ENV1-C, ENV1-T). Two cases were dated back to the Copper Age, both from Ossi, Sardinia. Four of the individuals who were positive for MMTVels were male, one female, and one probably female.

**Figure 3:**
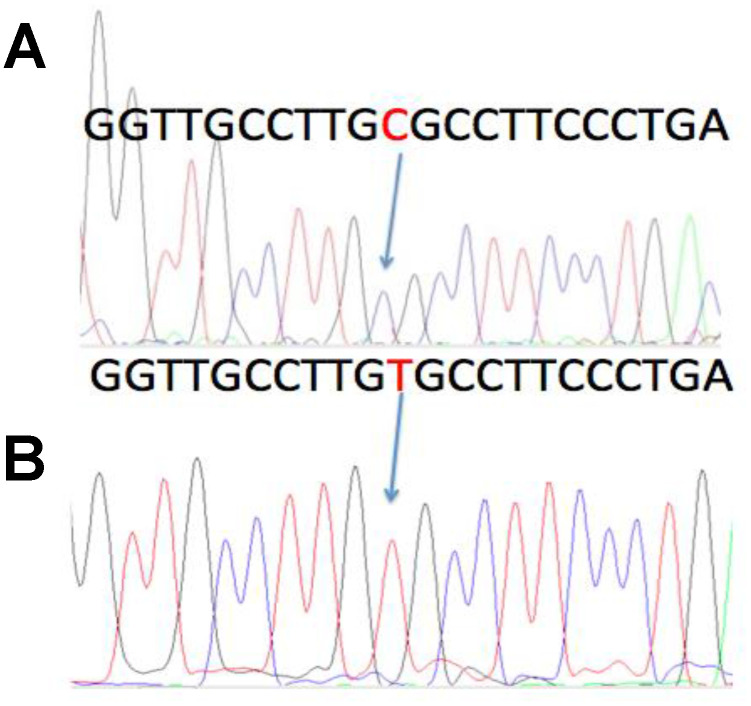
**A previously described C > T polymorphism in the MMTV DNA sequence.** In four of the MMTV positive cases, sequencing of both the ENV1 and ENV2 amplicons was accomplished. One of the ENV1 cases showed a previously described C > T polymorphism, that did not cause an amino acid substitution. The wild type sequence was named ENV1-C, whereas the polymorphic sequence was named ENV1-T. A: ENV1-C positive case from Luni, Liguria. B: The ENV1-T polymorphic case from Alghero, Sardinia.

Two different ENV regions were amplified, named herein ENV1 and ENV2 (Supplementary Figure 1). Six of the 36 cases examined were positive for ENV1, and two of them were also positive for ENV2 (Table 1). Two of the six positive cases dated back to the Sardinian Copper Age, approximately 4,500 years ago. The other four cases were from the 6^th^-17^th^ century. In four cases, both ENV1 and ENV2 were sequenced. One of the ENV1 amplicons had a C > T polymorphism (Figure 3), that has already been described and deposited in GenBank (accession number D16249.1), and does not cause an amino acid substitution. The ENV1 sequence with the T polymorphism was identified herein as ENV1-T, whereas the ENV1 wild-type sequence was identified as ENV1-C.

### The MMTV*env*-like sequences in ancient individuals are not an artifact of contamination

**Figure 4:**
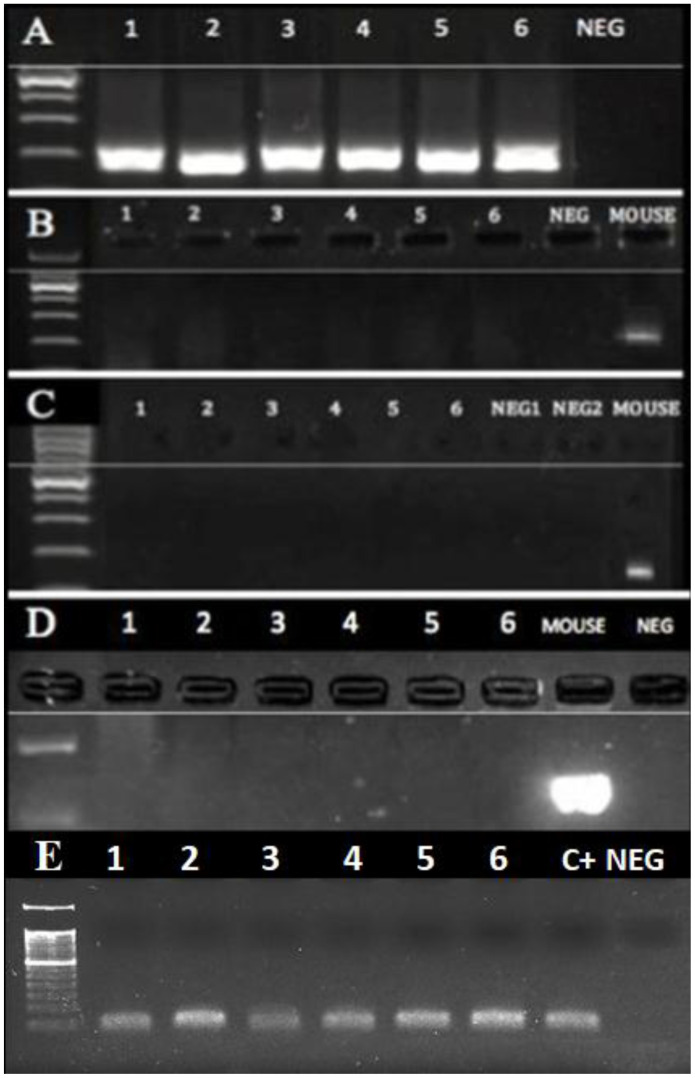
**PCR of DNA extracted from the dental calculus.** No mouse DNA was present in the six human cases (1-6) that were positive for MMTVels. (**A**) A 16S rRNA V3 region; all human cases were positive, whereas NEG was the negative control. (**B**) Intracisternal A Particle (IAP) LTRs; all the human cases were negative, as well as the negative control, whereas the mouse DNA was positive. (**C**) Murine mitochondrial DNA (mtDNA); all human cases were negative, whereas the mouse DNA was positive. (**D**) Murine GADPH; all the human cases were negative, whereas the mouse DNA was positive. (**E**) Human GADPH; all the human cases were positive; c+: a positive human control; neg: a negative control.

Mice are regular residents of tombs, where they often choose skulls as a nest. Hence, the presence of murine mitochondrial and intracisternal A particle DNA as well as the murine and human housekeeping gene GAPDH was assessed [26]. The results (Figure 4) showed that all samples positive for MMTVels contained neither murine mitochondrial DNA (Figure 4B) nor intracisternal A particle DNA (Figure 4C). Furthermore, the murine housekeeping gene GAPDH was not detected (Figure 4D), whereas human GAPDH was amplified (Figure 4E). To determine whether any DNA was present in our samples, they were PCR-amplified with a universal bacterial primer pair targeting the 16S rRNA (Figure 4A).

### Amplified MMTVels regions have high identity with previously described HMTV and MMTV *envs*

**Figure 5:**
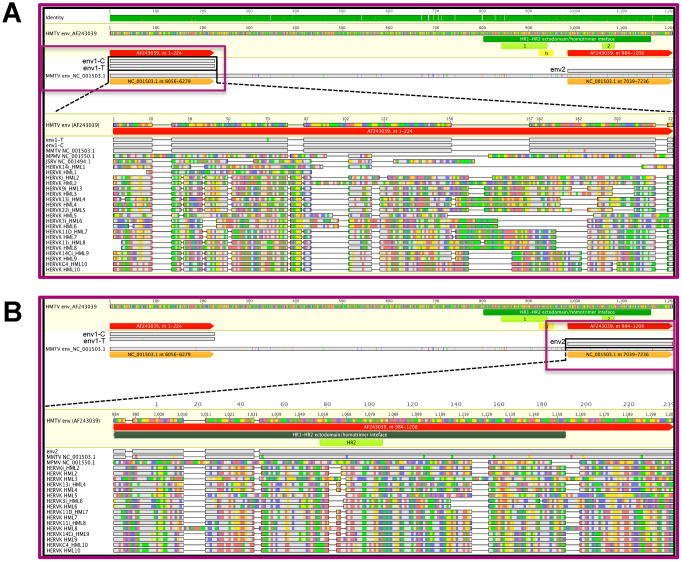
**Graphical representation of MMTV*env*-like amplicons compared to exogenous and endogenous betaretroviruses.** The ENV1-C/ENV1-T (panel **A**) and ENV2 (panel **B**) amplicons were mapped to the HMTV env reference sequence and compared with the latter and to other reference sequences representative for exogenous and endogenous betaretrovirus *env* gene in a multiple nucleotide alignment. For each sequence, grey bases represent residues that were identical to the reference HMTV, while colored residues indicate single nucleotide changes to A (red), C (blue), G (yellow), and T (green). All the three amplicons presented very few nucleotide substitutions with respect to MMTV (1 discordant nucleotide for ENV1-T and 0 for both ENV1-C and ENV2) and HMTV (2 discordant nucleotides for ENV1-T, 3 for ENV1-C and 11 for ENV2). The presence of predicted functional domains in HMTV portions corresponding or near the *env* amplicons is also annotated: Heptad Repeats 1 and 2 (HR1 and HR2, respectively) and immunosuppressive domain (IS). N.B. in the ENV2 alignment, JSRV is not present due to the absence of any shared nucleotide sequence.

To investigate the origin of DNA fragments obtained from dental calculus, the MMTV*env*-like amplicons were aligned with respect to the HMTV and MMTV sequences retrieved from public repositories (Supplementary Figure 2; see the Methods for details and accession numbers). Both ENV1-C and ENV1-T (224 bp) mapped to HMTV sequence at nucleotides 1-224 and to MMTV sequence at nucleotides 6056-6279, whereas ENV2 (225 bp) mapped to HMTV sequence at nucleotides 984-1208 and to MMTV sequence at nucleotides 7039-7236 (Figure 5). Alignment analysis showed that both ENV1-C and ENV2 shared 100% similarity with respect to the corresponding portions of HMTV *env*, whereas ENV1-T differed only at the polymorphic site. Conversely, all three amplicons showed a few single nucleotide differences compared to the corresponding portions of MMTV. In fact, ENV1-C and ENV1-T had 2 and 3 nucleotides differences respectively, whereas ENV2 harbored 11 different nucleotides (Figure 5 and Table 2). To assess the presence of functional domains, the obtained *env* amplicons were analyzed with the GenBank BLASTx tool to detect conserved motifs [26]. The results showed that the ENV2 amplicon was localized within HMTV Heptad Repeat 2 (HR2) region that, together with Heptad Repeat 1 (HR1), forms the HIV-1-like HR1-HR2 ectodomain, constituting the Env transmembrane portion fusion core (Figure 5).

**Table 2:** Pairwise nucleotide identity of the MMTV *env*-like amplicons with respect to exogenous and endogenous betaretroviruses.

	**ENV1-C**	**ENV1-T**	**ENV2**
***Exogenous betaretroviruses***			
HMTV AF243039	100·0% (0)	99·6% (1)	100·0% (0)
MMTV NC 001503.1	99·1% (2)	98·7% (3)	95·1% (11)
MMTV C3H AF228552.1	52·5% (125)	52·5% (125)	94·3% (13)
JSRV (sheep)	27·9% (150)	27·9% (150)	-
MPMV (monkey)	22·6% (181)	22·6% (181)	20·1% (191)
***Endogenous betaretroviruses***			
HERVK14i HML1	29·2% (126)	29·2% (126)	-
HERVK HML1*	28·9% (32)	28·9% (32)	-
HERVKi HML2	28·9% (170)	28·9% (170)	33·8% (151)
HERVK HML2*	26·2% (200)	26·2% (200)	35·5% (147)
HERVK9i HML3	33·6% (156)	33·6% (156)	-
HERVK HML3*	33·9% (156)	33·9% (156)	32·5% (154)
HERVK13i HML4	30·0% (161)	30·0% (161)	30·7% (158)
HERVK HML4*	36·1% (147)	36·1% (147)	34·2% (150)
HERVK22i HML5	33·7% (156)	33·7% (156)	-
HERVK HML5*	25·7% (168)	25·7% (168)	25·7% (171)
HERVK3i HML6	27·5% (169)	27·5% (169)	30·0% (161)
HERVK HML6*	28·8% (166)	28·8% (166)	31·7% (157)
HERVK11D HML7	31·3% (158)	31·3% (158)	35·5% (147)
HERVK HML7*	30·9% (159)	30·9% (159)	35·1% (148)
HERVK11i HML8	30·0% (161)	30·0% (161)	35·5% (147)
HERVK HML8*	29·6% (162)	29·6% (162)	35·4% (153)
HERVK14Ci HML9	31·7% (157)	31·7% (157)	32·5% (154)
HERVK HML9*	32·2% (156)	32·2% (156)	32·9% (153)
HERVKC4 HML10	21·4% (167)	21·4% (167)	32·9% (153)
HERVK HML10*	27·4% (167)	27·4% (167)	33·3% (152)

The pairwise comparison between the identified MMTVels and other exogenous and endogenous betaretroviruses showed that all the amplicons shared a very high identity with MMTV and its putative human homologous HMTV, being instead highly divergent with respect to the other exogenous and endogenous betaretroviruses. Identity values are expressed as % of identical nucleotides. The number of divergent nucleotides is also indicated between brackets. Consensus sequences marked with * are from [18].

### Amplified MMTVels do not derive from HERV-K HML amplification

**Figure 6:**
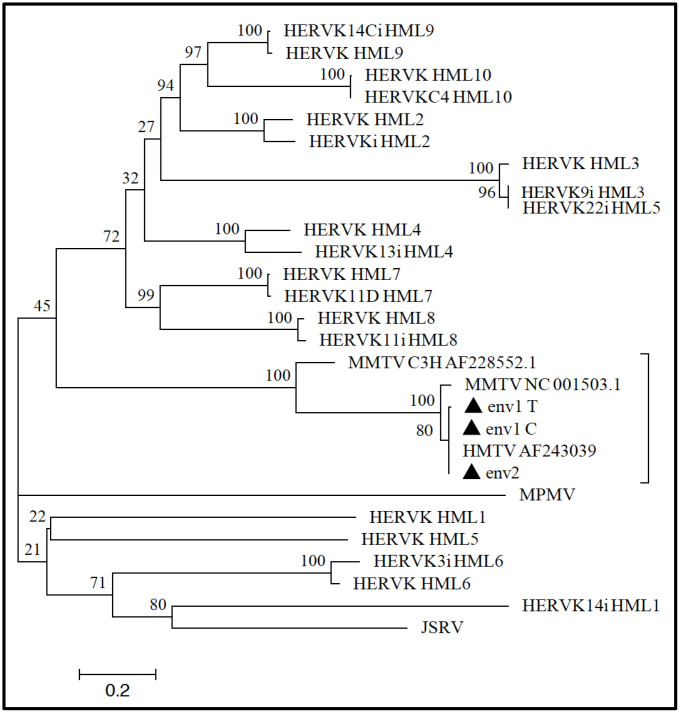
**Phylogenetic analysis of MMTV *env*-like amplicons.** ENV1-C, ENV1-T, and ENV2 amplicons (filled triangles) were analyzed to infer their phylogeny with respect to the other exogenous and endogenous betaretrovirus nucleotide sequences (see Materials and Methods for further details). The close relationship between the tree MMTVels amplicons and both MMTV and HMTV was confirmed at the phylogenetic level, given that all the sequences grouped together in the same clade that was statistically supported by the maximum bootstrap value. The amplicons were instead not related to any of the HERV-K HML groups’ sequences, which were clearly clustered in a different phylogenetic clade. Evolutionary relationships were inferred by using the maximum likelihood method and the Kimura-2-parameter model, and the resulting phylogeny was tested using the Bootstrap method with 100 replicates. The length of branches indicates the number of substitutions per site.

Given the high degree of similarity between MMTV and HERV-K HML integrations, we examined whether the MMTVels fragments could have originated from the amplification of human endogenous sequences. The three MMTV*env*-like amplified fragments were aligned with the consensus sequences of HERV-K HMLs 1 to 10, based on the Repbase Update repository [27] and the database published by Vargiu et al. [18] as well as with MMTV reference sequence, the MMTV strains C3H, JSRV, and MPMV, and the proposed HMTV reference sequence (see the Material and Methods for further details and accession numbers). Comparison of the ENV1-C and ENV1-T amplicons was possible with all the considered betaretroviruses, while the ENV2 amplicons showed no sequence correspondence in the HERV-K HML1, HML3, and HML5 Repbase consensuses or the JSRV reference sequence (Figure 6).

The results showed that all three MMTV*env*-like amplified fragments shared overall very low identity (26% to 36%) with respect to all the HERV-K HML consensus sequences (Table 2). In contrast, the analysis demonstrated that all the amplicons shared remarkable sequence similarity with the HMTV and MMTV consensus sequences (Table 2). Particularly, both ENV1-C and ENV1-T showed high nucleotide identity with HMTV and MMTV (100% and 99.6%, respectively) and lower identity with the MMTV C3H strain (52.5%), while the ENV2 amplicon was highly identical to HMTV, MMTV, and the MMTV CH3 strain, with percentages of shared nucleotides of 100%, 95% and 94%, respectively (Table 2). In addition, all three MMTV*env*-like amplicons showed high divergence compared to JSRV and MPMV exogenous betaretroviruses, with only 20% to 28% identity (the comparison between JSRV and the ENV2 amplicon was not possible due to the complete absence of a shared nucleotide sequence between the two sequences) (Table 2 and Figure 6). Overall, the above results exclude the possibility that the MMTV*env*-like amplicons obtained from ancient dental calculus may have derived from betaretroviral HERV amplifications.

### Phylogenetic analysis of the MMTV*env*-like amplified regions

The alignment built to analyze the ENV amplicons nucleotide identity with other betaretroviruses was also used to infer their phylogeny with respect to the same exogenous and endogenous elements (Figure 6). Maximum likelihood analysis showed that all three MMTV*env*-like amplicons grouped together with HMTV and MMTV, as supported by the highest bootstrap value (=100). This group was also significantly related to the MMTV C3H strain (bootstrap support=100), but it was clearly phylogenetically distinct from all the other exogenous and endogenous betaretroviruses (Figure 6), as suggested by the low nucleotide identity.

## DISCUSSION

Betaretroviruses are well-known pathogenic agents in animal models, such as Mason-Pfizer Monkey Virus (MPMV), Mouse Mammary Tumor Virus (MMTV), and Jaasiekte Sheep Retrovirus (JSRV), which cause immunodeficiency in macaque [28], mammary tumors in mouse [6] and pulmonary adenocarcinoma in sheep, respectively [29]. Contrarily, no human exogenous betaretrovirus is recognized as a causative agent in human diseases, given that, actually, no human exogenous betaretrovirus is known.

The strong similarity between human breast cancer and murine mammary tumors raised the question of the potential role of a betaretrovirus in breast carcinogenesis. In the 90s, molecular techniques allowed the detection of MMTVels in a high percentage of human infiltrating breast carcinomas [8, 9]. The authors suggested that the presence of such sequences was indicative of a human MMTV-like virus, named HMTV. Accordingly, a very recent review details all the data in favor of the viral etiology of human breast carcinoma [3]. Here we limit our discussion to the following: a) MMTV *env* sequences are absent in the human genome, whereas they are present in breast tumors and in normal breast tissues [30]; b) MMTVels have been identified in breast tissues prior to the development of MMTVels-positive breast cancer [31]; c) MMTVels have been detected in a high percentage of pre-invasive BC lesions, such as atypical epithelial hyperplasia and ductal carcinoma *in situ* (DCIS) [32]; d) the percentage of positivity of DCIS (80%) is double than that of the infiltrative tumors, which is confirmed to be between 30% and 40% [32]; and e) chromogenic *in situ* hybridization experiments have demonstrated the presence of viral hybridization signal in tumor nuclei, with a 50% reduction in infiltrating tumors compared to DCIS [32]. The strong reduction of positive cases moving from *in situ* to infiltrating tumors is of particular interest because it could indicate a possible relevance for the virus in cell transformation only and not for cancer progression. This phenomenon could be a consequence of DNA loss owing to the high level of chromosomal rearrangement characterizing breast tumors. Two findings are of special relevance and deserve a comment: the almost complete absence of MMTV sequences in hereditary breast carcinomas [1] and their presence in human salivary glands and saliva [16].

The difference in the results between sporadic tumors (with a high percentage of positive cases, 30.3%) and hereditary tumors (with a very high percentage of negative cases, almost 96%) is too distinct (*p* < 0.001) for it to be a mere coincidence or the consequence of a contamination with murine material, and does not allow to abandon the hypothesis of a viral etiology for human breast carcinoma. Moreover, the p14 protein, the signal peptide of the MMTV envelope precursor [33], is expressed only in cases that are positive for MMTVels [1].

The analysis of saliva was suggested by the fact that there are biological reasons that make milk improbable as a vehicle in MMTV-like virus transmission in humans [16] and that in humans a long duration of lactation is considered protective against breast cancer or ineffective [34]. The presence of MMTVels in saliva, suggesting the existence of a human MMTV-like betaretrovirus, inspired the present study.

We investigated the presence of MMTVels in ancient dental calculus, an important source for analyses of the paleomicrobiome and paleovirome. Saliva, in fact, provides many constituents of dental plaque, a bacterial biofilm covering the surface of teeth. Over time, plaque undergoes a process of calcification, giving origin to the dental calculus (tartar) through gradual calcium phosphate mineralization, in which all bacteria and viruses are entombed, providing excellent preservation of nucleic acids [35]. Dental calculus is commonly found in humans, both modern and ancient, including Neanderthals [36]. Paleomicrobiology has benefitted from the advances in high-throughput biomolecular sequencing, which can be easily applied to the analysis of calculus, which is considered an abundant and long-term reservoir of the ancient oral microbiome [35]. It must be stressed that the calculus is not easily colonized by environmental bacteria, does not have points of entry for exogenous bacteria, and lacks nutrient sources that are able to attract and support bacteria growth [37].

The study of dental calculus from 36 ancient skulls revealed MMTVels in 6 of them, of which two were from the Copper Age, i.e. 4,500 years ago, and 4 were from the 15^th^-17^th^ century. The three identified sequences corresponded to the MMTV *env* gene and two of them differed only for an already described single nucleotide C>T polymorphism, being identified herein as ENV1-C, ENV1-T and ENV-2. We focused on the *env* gene because it is able to transform human epithelial mammary cells in culture and, for this reason, it has been object of all the previous studies concerning MMTV in human material [38].

The existence of a high number of Human MMTV-like (HML) sequences in the human genome over millions of years made it crucial to exclude the possibility that our *env* sequences derived from the amplification of HERVs. In fact, due to their abundance and remarkable similarity with MMTV, HML sequences can lead to misinterpretation when investigating the occurrence of exogenous betaretroviruses in humans, possibly leading to false positives.

The careful bioinformatics analysis conducted herein demonstrated that the MMTVels identified in the ancient calculus did not have a HERV origin. In fact, the amplified ENV sequences were aligned and compared with the corresponding sequences of the exogenous animal betaretroviruses and the HERV-K HML 1 to 10 group consensus sequences, showing very high identity to HMTV and MMTV and, in contrast, very low identity to the other animal exogenous betaretroviruses and to all HERV-K HMLs. In addition, the phylogenetic analysis demonstrated that the amplified *env* regions were significantly related to HMTV, MMTV and MMTV C3H, whereas they were quite distinct from all the other exogenous and endogenous betaretroviruses.

Overall, our study adds a piece to a puzzling scenario, which includes a number of scientists that are very skeptical about the hypothesis of a viral etiology of breast cancer and believe that all the positive results obtained over many decades are only a consequence of contamination with murine material or are due to the presence of endogenous betaretroviral sequences. This motif, even with dubious and inconclusive positions, has become recurrent, reducing the consideration of numerous biological and molecular data that tentatively link MMTV to human breast cancer, as summarized above and elsewhere [3]. However, the almost complete absence of MMTVels in hereditary breast carcinomas [1], the careful exclusion of possible contamination by murine material and bacteria [1, 26, 37], and the demonstration that the sequences identified in the present study do not have endogenous origin, do not allow to simply refuse such hypothesis, calling for further studies to finally demonstrate the possible existence of a modern human betaretrovirus.

## CONCLUSIONS

This paper reveals that betaretroviral sequences sharing high identity with MMTV have been present in human species for at least 4,500 years.

This observation, together with all previous data concerning MMTV in humans, suggests the existence of a human exogenous betaretrovirus possibly derived from a cross-species transmission that occurred in prehistoric times, with consequent inter-human spread.

It is worth to note that mice became commensals of humans approximately 10,000 years ago, at the beginning of civilization, with the rise of agriculture in the area named the fertile crescent [39], which includes Western Asia, the Nile Valley, and the Nile Delta, guaranteeing mice an abundance of food, and giving origin to a period of strict cohabitation between the two species. In fact, humans also began to cohabit with other species, including those that they learned to domesticate. This situation played a relevant role in cross-species transmission – or species jump – with the evolution of animal microorganisms into human pathogens. An important example of cross-species transmission is represented, among others, by the human immunodeficiency virus (HIV), which has passed from chimpanzees to humans [40].

The data described herein strongly support the further investigation of the role of a MMTV-like element in human breast cancer. In this case, prophylactic strategies could be established based on the development of a specific vaccine or an immunotherapeutic approach. Interestingly, the immune-mediated targeting of MMTV-p14 protein has been recently proposed [41].

## MATERIALS AND METHODS

### Study design

Dental calculus was collected from 36 skulls from the Copper Age to 17^th^ century. Total DNA was extracted from calculus and whole genome amplification was performed. MMTVels were detected by fluorescence-nested PCR. Attention was focused on the *env* gene due to its ability to transform human mammary epithelial cells in culture [38]. Contamination by mouse DNA was excluded. The identified sequences were compared to endogenous betaretroviruses to assess their origin.

### Characterization of the individuals

The dating of the a.C. individuals was performed by ^14^C and on the basis of archaeological and historical information for the A.D. cases. The two oldest cases (2560 ± 51 BC) belonged to the Copper Age (Chalcolithic, Eneolithic), which includes the period from approximately 3700/3400 BC to 2300 BC [42, 43]. The Copper Age was an archeological era characterized by the use of native copper for the production of metal tools. The more recent Bronze Age begins when humans discovered that adding tin to copper gave origin to the harder and stronger bronze.

Sex was determined according to Ferembach et al., whereas age at death was assessed by the sternal rib end modification and by dental wear [44, 45].

### Collection of calculus

Operators wore a mask and sterilized gloves and used sterilized scalpels. New gloves and scalpels were used for each skull. Calculus was collected in sterilized tubes.

### DNA extraction

Approximately 50 mg of dental calculus was crushed in a 2 ml tube and digested overnight at 56°C in 1 ml 0.45M EDTA with 10% proteinase K (Promega, Madison, WI, USA), followed by a 24 hours digestion at room temperature on an agitator. The entire volume was loaded in an automated system Maxwell 16 system (Promega, Madison, WI, USA) using the Maxwell® 16 LEV DNA Purification Kit.

### Whole-genome amplification

Samples were subjected to whole-genome amplification using the Genomeplex Single Cell Whole Genome Amplification kit (Sigma-Aldrich, Saint Louis, MO, USA) starting from 7 μl of DNA and following the supplied protocol. The amplified DNA was measured with a Qubit 2.0 Fluorometer (Invitrogen, Life Technologies, Grand Island, NY) using the Qubit DNA assay kit to obtain a concentration ranging from 30 to 90 ng/μl per sample.

### PCR housekeeping gene and murine contamination analysis

To determine whether any DNA was present in our samples, DNA extracts were PCR-amplified with a universal bacterial primer pair targeting the 16S rRNA V3 region: forward, 5’-ACTCCTACGGGAGGCAGCAGT-3’, reverse, 5’-GTATTACCGCGGCTGCTGGCAC-3’ [46].

The presence of contaminating mouse DNA was excluded by performing murine mitochondrial DNA and IAP LTRs PCR [25] and murine GAPDH PCR.

### Detection of MMTVels: PCR and sequencing analysis

Fluorescence-nested PCR was used to detect the presence of DNA MMTVels. Two sets of primers were designed to amplify two different regions, ENV-1 and ENV-2 (Supplementary Figure 1), based on the sequence available in GenBank with accession number AF243039. ENV-1 was amplified by semi-nested 224-155 bp PCR using the following primers: ENV1Forward 5’-ccagatcgcctttaagaagga-3’, ENV1Reverse 3’-actccccctgtcaataaagagg-5’, and ENV1Reverse 3’-ccttccctcgcctagtgtag-5’. For amplification of ENV-2 (225 bp), we used a nested-PCR with the following primers: ENV2Forward 5’-ctgagagctgggaaagaacc-3’ and ENV2 Reverse 3’-ctgagagctgggaaagaacc-5’. Both ENV-1 and ENV-2 PCRs were carried out using a previously described protocol [32].

The products of PCR amplification were sequenced, after clean-up with the QIAquick PCR Purification Kit (Qiagen, Venlo, Netherlands), using Big Dye Terminator mix (Applied BioSystems, Warrington, UK). Sequencing reactions were run on an ABI3130 XL (Applied BioSystems, Warrington, UK).

### Sequences considered in the study

To characterize their virological origin, the MMTVels amplicons were compared to the publicly available exogenous and endogenous betaretroviral reference/consensus sequences:

- *Exogenous animal betaretroviruses:* MMTV (NC001503), MMTV C3H strain (AF228552.1), JSRV (NC001494.1), MPMV (NC001550.1).
- *HMTV* (AF243039, *env*-LTR portion), the original MMTV identified in human breast tumors by Pogo et al. [8].
- *Endogenous human betaretroviruses:* HERV-K HML 1 to 10 based on the RepBase Update database and on the recently provided dataset of ~3200 HERV insertions [18, 27].

### Bioinformatics analysis

The above nucleotide sequences and the corresponding amino acid translation have been aligned and compared regarding their identity and phylogenetic relationships as follows:

*Sequences alignment:* multiple alignments and their graphical depictions were generated using the Geneious bioinformatics software platform, version 8.1.4 with the MAFFT algorithms FFT-NS-i x1000 or G-INS-I with default parameters [47, 48]. All multiple alignments were visually inspected and, when necessary, manually optimized prior to subsequent analyses.

*Phylogenetic analysis*: the sequence phylogenetic analysis was performed by using the Maximum Likelihood (ML) method with the MEGA Software, version 6, based on the Kimura 2-parameter model [49]. Phylogenies were tested by the bootstrap method with 100 replicates. Pairwise deletion option was applied, allowing to compare only the nucleotide sequences of portions that were actually shared by the sequences. The tree was drawn to scale, with branch lengths measured as the number of substitutions per site.

*Nucleotide divergence estimation*: pairwise divergences between aligned nucleotide sequences were estimated using MEGA Software, version 6 [49], with a p-distance model and applying the pairwise deletion option**.**

## Supplementary Material

Supplementary Figures
