# Supplementary material for: A human MMTV-like betaretrovirus linked to breast cancer has been present in humans at least since the copper age
Source: Aging (Albany NY). 2020 Jul 31;12(16):15978–94. doi: 10.18632/aging.103780 (PMC7485742; doi:10.18632/aging.103780)
Supplement: Supplementary Figures [file aging-12-103780-s001..pdf]

SUPPLEMENTARY FIGURES

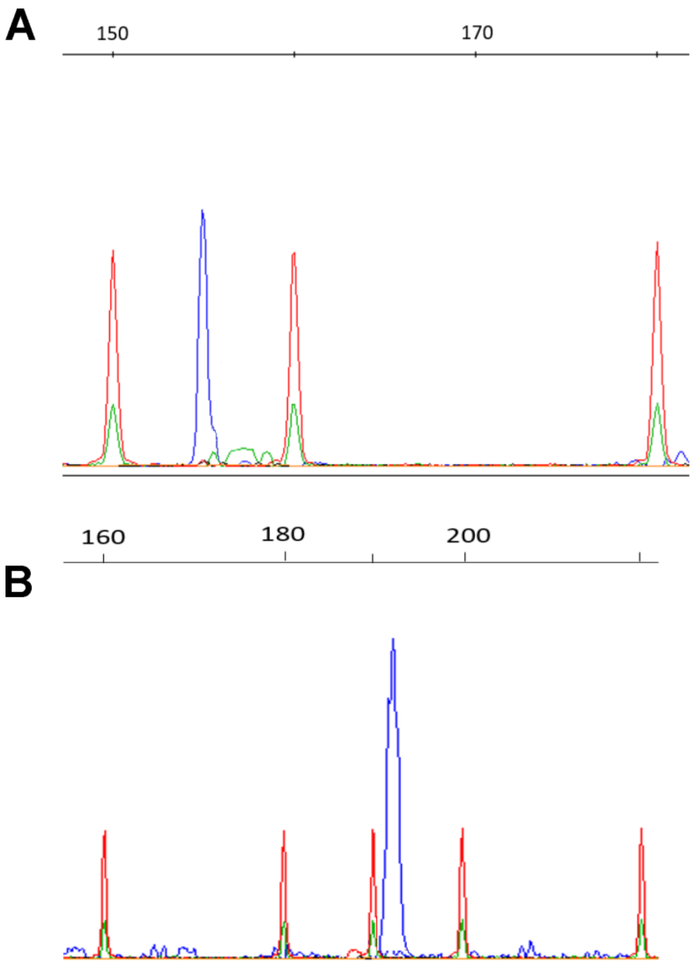

Supplementary Figure 1. Electropherograms produced by capillary gel electrophoresis of amplified DNA products ENV1 and ENV2. After nested PCR the final PCR product measures 155bp for ENV1 (**A**) and 191bp for ENV2 (**B**).

```
>MMTV_
CCAGATCGCCTTTAAGAAGGACGCCTTCTGGGA-----------------------
GGGAGACGAGTCTGCTCCTCCACGGTGGTTGCC-
TTGCGCCTTCCCTGACCAGGGGGTGAGTTTTTCTCCAAAAGGGGCCCTT-------------------------
----------------
GGGTTACTTTGGGATTTCTCCCTTCCCTCGCCTAGTGTAGATCAGTCAGATCAGATTAAAAG------------
-------
CAAAAAGAATCTATTTGGAAATTATACTCCCCCTGTCAATAAAGAGGTTCATCGATGGTATGAAGCAGGATGGG
TAGAACCTACTTGGTTCTGGGAAAATTCTCCTAAGGATCCCAATGATAGAGATTTCACTGCACTAGTCCCC---
CATACAGAATTGTTTCGCTTAGTCGCAGCCTCAAGACATCTTA-------------------------------
--------------------------------------------------------------------------
--------------------------------------------------------------------------
--------------------------------------------------------------------------
----------------------------------------------------------
TTCTCAAAAGGCCAGGATTTCAAGAACATGAGATGATTCCTACATCTGCCTGTGTTACTTACCCTTATGCCATA
TTATTAGGATTACCTCAGTTAATAGATAT---AGAGAAAAGAGGATCTA--
CTTTTCATATTTCCTGTTCTTCTTGTAGATTGAC-------------
TAATTGTTTAGACTCTTCTGCCTACGACTATGCAGCGATCATAGTCAAGAGGCCGCCATATGTGCTGCTACCTG
TAGATATTGGTGATGAACCATGGTTTGATGATTCTGCCATTCAAACCTTTAGGTATGCCA--------------
-----------------------CAGATTTAATTCGAGCTAAGCGATTCGTCGCA----
GCCATTATCCTGGGCATATC-TGCTTTGATTGCTATTATTACTTCCT----TTGCTG---------
TAGCTACTACTGCCTTAGTTAAGGAGATGCAAACTGCTACGTTTGTTAATAATCTTCATAGGAATGTTACAT--
---------------------
TAGCCTTATCTGAACAACGGATAATAGATTTAAAATTAGAAGCTAGACTTAATGCTTTAGAAGAAGTAGTTTTA
GAGTTG--------------------
GGACAAGATGTGGCCAATTTAAAGACCAGAATGTCCACTAGGTGTCATGCAAATTATGAC--
TTTATCTGCGTTACAC----------------CTTTACCCTATAATGCTACTGAGAAC---
TGGGAAAGAACCAGGGCTCATTTATTGGGCATTTGGAATGATAATGAGATTTCATATAACATACAGGAGTTAAC
CAA----------CCTGATTAGTGATA---TGAGCAAACAACAT-------------------
ATTGATGCAGTGGACCTTAGTGGCTTGGC-TCAGTCTTTTGCCA--ATGGAGTGAAGGCTTTA------
AATCCATTAGATTGGACAC--AATATT----------------------------------
TCATTTTTATAGGTGTTGGAGCCCTGC
>HMTV
CCAGATCGCCTTTAAGAAGGACGCCTTCTGGGA-----------------------
GGGAGACGAGTCTGCTCCTCCACGGTGGTTGCC-
TTGCGCCTTCCCTGACCAGGGGGTGAGTTTTTCTCCAAAAGGGGCCCTT-------------------------
----------------
GGGTTACTTTGGGATTTCTCCCTTCCCTCGCCTAGTGTAGATCAGTCAGATCAGATTAAAAA------------
-------
CAAAAAGGATCTATTTGGAAATTATACTCCCCCTGTCAATAAAGAGGTTCATCGATGGTATGAAGCAGGATGGG
TAGAACCTACTTGGTTCTGGGAAAATTCTCCTAAAGATCCCAATGATAGAGATTTTACTGCTCTAGTTCCC---
CATACAGAATTGTTTCGCTTAGTTGCAGCCTCAAGACATCTTA-------------------------------
--------------------------------------------------------------------------
--------------------------------------------------------------------------
--------------------------------------------------------------------------
----------------------------------------------------------
TTCTCAAAAAGCCAGGATTTCAAGAACGTGAGATGATTCCTACATCTGCCTGTGTTACTTACCCTTATGCCATA
TTATTAGGATTACCTCAGCTAATAGATAT---AGAGAAAAGAGGATCTA--
CTTTTCATATTTCCTGTTCTTCTTGTAGATTGAC-------------
TAATTGTTTAGATTCTTCTGTCTACGACTATGCAGCGATCATAGTCAAGAGCCGGCCATATGTGGTGCTACCTG
TAGATATTGGTGATGAACCATGGTTTGATGATTCTGCCATTCAAACCTTTAGGTATGCCA--------------
-----------------------CAGATTTAATTCGAGGTGAGCGATTCGTCGGT----
GCCATTATTGTGGGCATATA-TGCTTTAATTGGTATTATCACTTCCT----TTGCTG---------
TAGGTACTACTGCCTTAGTTAAGGAGATGCAAACTGCTACGTTTGTTAATAATCTTCATAGACATGTTACAT--
---------------------
TAGCCTTATCTGAACAAAGAATAATAGACTTTAAATTGAAAGTTAGACTTAATGCTTTAGAAGAAGTAGTTTTA
GAGTTG--------------------
GGACAAGATGTGGCAAACTTAAAGACCAGAATGTCCACTAGGTGTCATGCAAATTATGAT--
TTTATCTGTGTCACAC----------------CTTTGCCATATAATGCTTCTGAGAGC---
TGGGAAAGAACCAGAGCTCATTTATTGGGCATTTGGAATGACAATGAGATTTCATATAACATACAGGAGTTAAC
CAA----------TCTGATTAGTGATA---TGAGCAAACAACAT-------------------
ATTGACGCAGTGGACCTCAGTGGCTTGGC-TCAGTCCTTTGCCA--ATGGAGTAAAGGCTTTA------
AATCCATTGGATTAGACAC--AATATT----------------------------------
TCATTTTTATAGGAGTTGGAGCCCTGC
```

Supplementary Figure 2. Fasta nucleotide alignment of the identified MMTV-like env amplicons with human endogenous betaretroviruses and representative animal exogenous betaretroviruses (MMTV, murine mammary tumour virus; JSRV, Jaasiekte Sheep Retrovirus; MPMV, Mason-Pfizer Monkey Virus).
